# Hemoglobin catalyzes CoA degradation and thiol addition to flavonoids

**DOI:** 10.1038/s41598-018-19585-7

**Published:** 2018-01-19

**Authors:** Toshiki Nagakubo, Takuto Kumano, Yoshiteru Hashimoto, Michihiko Kobayashi

**Affiliations:** Graduate School of Life and Environmental Sciences, University of Tsukuba, 1-1-1 Tennodai, Tsukuba, Ibaraki, 305-8572 Japan

## Abstract

In the presence of CoA, cell-free extracts prepared from porcine liver was found to convert 7,8-dihydroxyflavone (DHF) to a pantetheine conjugate, which was a novel flavonoid. We purified a 7,8-DHF-converting enzyme from the extracts, and identified it as hemoglobin (Hb). The purified Hb showed the following two activities: (*i*) degradation of CoA into pantetheine through hydrolytic cleavage to yield pantetheine and 3′-phospho-adenosine-5′-diphosphate (ADP) independently of heme, and (*ii*) addition of a thiol (e.g., pantetheine, glutathione and cysteine) to 7,8-DHF through C-S bond formation. Human Hb also exhibited the above flavonoid-converting activity. In addition, heme-containing enzymes such as peroxidase and catalase added each of pantetheine, glutathione and cysteine to the flavonoid, although no pantetheine conjugates were synthesized when CoA was used as a substrate. These findings indicated that the thiol-conjugating activity is widely observed in heme-containing proteins. On the other hand, only Hb catalyzed the hydrolysis of CoA, followed by the thiol conjugation to synthesize the pantetheine conjugate. To the best of our knowledge, this is the first report showing that Hb has the catalytic ability to convert naturally occurring bioactive compounds, such as dietary flavonoids, to the corresponding conjugates in the presence of thiol donors or CoA.

## Introduction

For many years, natural products have been recognized as a rich source of compounds for drug discovery. Various kinds of antibiotics and bioactive compounds were discovered from natural resources^1^. In many countries, on the other hand, chronic diseases such as cancer, heart disease, and stroke remain considerable public concerns. Traditionally, a plant-based diet, which usually contains various flavonoids, is thought to reduce the risk of these diseases and to promote health. Thousands of flavonoids have been isolated from plants. Plants synthesize flavonoids to protect themselves from various environmental stressors such as harmful ultraviolet (UV) radiation and oxidative species^2^.

A number of epidemiological studies suggested that flavonoids in vegetables and fruits protect humans against chronic diseases^3^. Inhibitory effects on the activities of mammalian enzyme systems and the free radical-scavenging activities of flavonoids contribute to the protection against such diseases^4^. The ingested flavonoids in humans are metabolized through two pathways, as follows. (i) In the small intestine and liver, flavonoids are glucuronidated, sulfonated and *O*-methylated by UDP-glucuronosyltranferase, sulfotransferase and catechol-*O*-methyltransferase, respectively^5^. (ii) Intestinal bacteria degrade flavonoids into a variety of hydroxylated phenyl carboxylic acids^5^. Flavonoids absorbed by the small intestine are exclusively converted into conjugates in the liver, and the resultant conjugates enter the circulatory system^6^. These conjugates are mostly deconjugated in tissues. The resulting flavonoid aglycones are considered to have biological functions in the cell^7^. However, it is also proposed that the conjugated metabolites themselves show antioxidative activities and inhibitory effects on the activities of some mammalian enzymes^8^. For example, quercetin and its glucuronidated metabolite inhibited myeloperoxidase, which catalyzes low-density lipoprotein oxidation^9^. Also, an *O*-methylated flavonoid inhibited the activity of NADPH oxidase, which generates reactive oxygen species (ROS) in the inflammation process^10^. Moreover, glucuronidated and sulfated flavonoids showed protective activity against endothelial dysfunction *in vivo*^11^. These unique activities of metabolized flavonoids are a part of the bioactivity of ingested flavonoids. Therefore, identification of a novel metabolite of a flavonoid and a flavonoid-converting enzyme is essential for understanding of the functions of dietary flavonoids.

**Figure 1:**
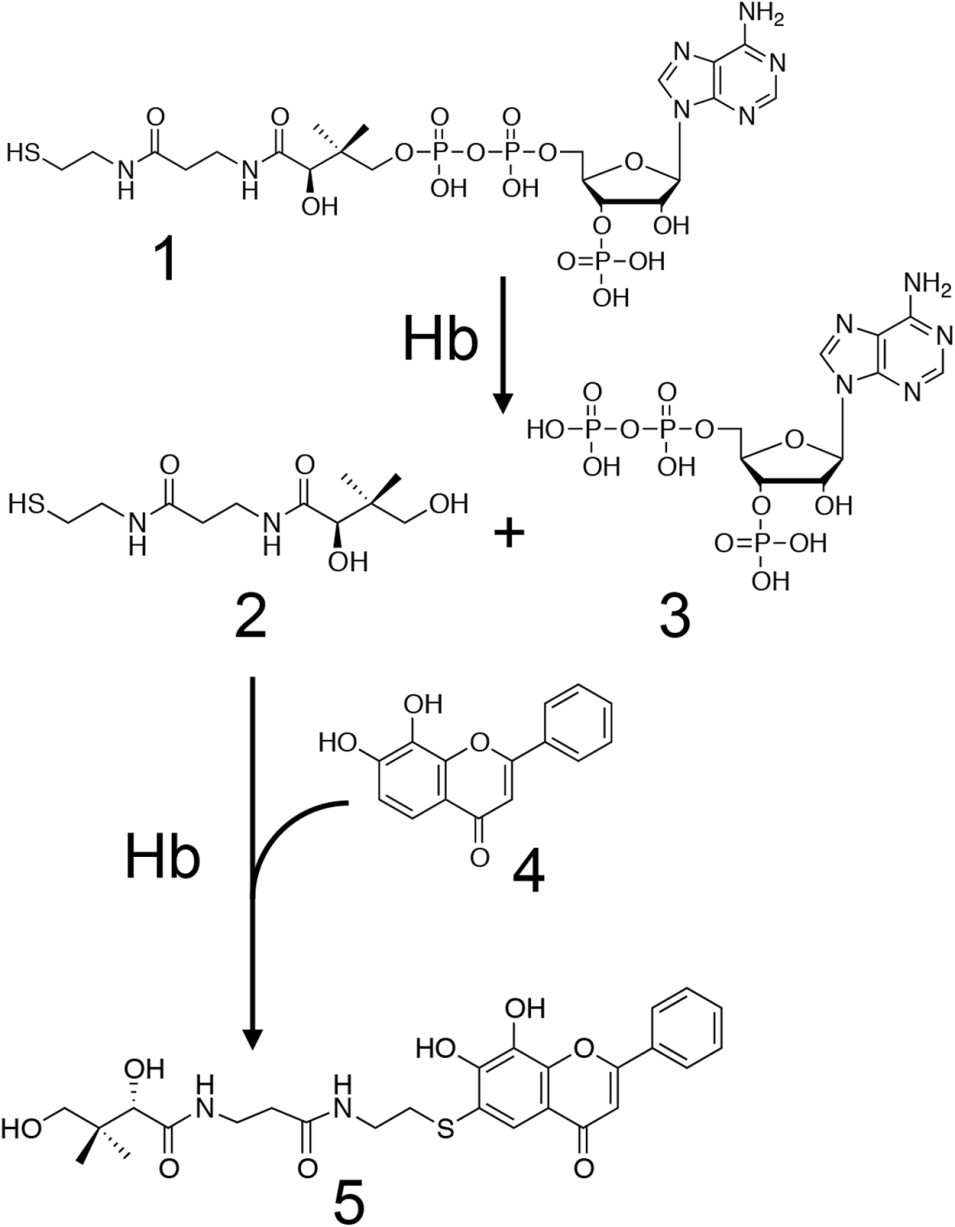
The reaction scheme for the formation of pantetheine-conjugated 7,8-DHF by Hb. The compounds in this figure are as follows; CoA (**1**), pantetheine (**2**), 3′-phospho-ADP (**3**), 7,8-DHF (**4**), pantetheine-conjugated 7,8-DHF (**5**).

In our laboratory, recently, we have studied the metabolism of biologically active natural compounds, such as curcumin^12^, sesamin^13^, and piperonal^14^. Very recently, particularly, we proposed a new biocatalyst concept; actinorhodin, a natural low-molecular mass organic compound, acts as a biocatalyst under physiological conditions^15^. This finding opens the door to a new field of organocatalysts in living organisms. In the present article, we show that heme-containing catalysts including hemoglobin (Hb), peroxidase and catalase add each of thiols (e.g., pantetheine, glutathione and cysteine) to flavonoids. Moreover, we report that Hb has the ability to cleave CoA to yield pantetheine and 3′-phospho-ADP (Fig. 1). We propose a new function of Hb.

## Results

### Detection of a novel metabolite of 7,8-dihydroxyflavone

**Figure 2:**
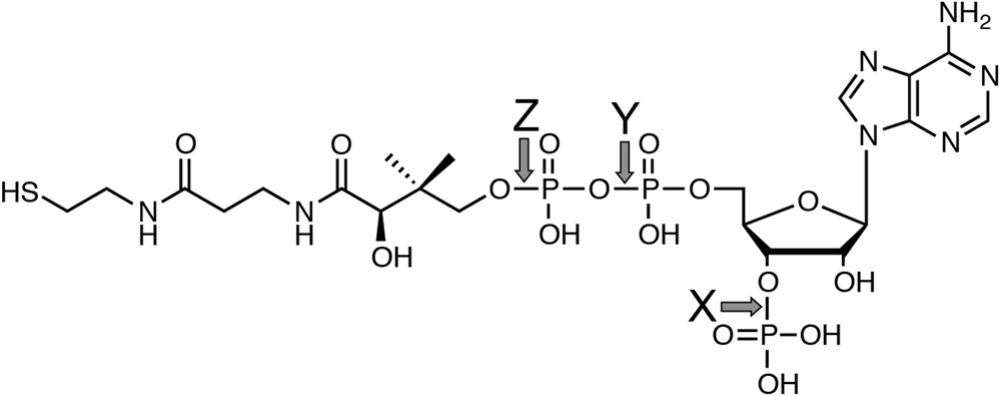
The structure of CoA. Arrows *X*, *Y*, and *Z* indicate the cleavage sites in CoA for CoA phosphatase, dephospho-CoA pyrophosphatase and Hb, respectively.

In order to detect and identify a novel metabolite of a flavonoid, we incubated cell-free extracts prepared from the liver of a pig with 7,8-dihydroxyflavone (DHF) for 10 h at 37 °C. After incubation of the reaction mixture, however, no reaction product was observed on LC/MS analyses. Next, we added each of AMP, ADP, ATP, NADH, NADPH, NAD^+^, NADP^+^, FAD, FMN, inositol, thiamine·HCl, pantothenic acid, vitamin B_6_, and CoA (Fig. 2) to the above reaction mixture at a final concentration of 1 mM. After incubation for 7 h at 37 °C, the products that could be derived from 7,8-DHF were formed when CoA was added to the reaction mixture (Fig. S1). One of the reaction products gave a [M-H]^−^ ion at *m*/*z* 529.1631, which is in good agreement with the molecular formula [C_26_H_29_N_2_O_8_S]^−^. This molecular formula corresponded with that of the pantetheine conjugate of 7,8-DHF. Unexpectedly, these results indicate that CoA was used as a substrate but not as a cofactor, being degraded into pantetheine and adducted to 7,8-DHF by a 7,8-DHF-converting enzyme.

### Purification and identification of the 7,8-DHF-converting enzyme

Through the purification steps described under “*Methods*”, the 7,8-DHF-converting enzyme was purified over seven-fold, with a yield of 0.230%, from the liver of a pig (Table S1). The purified enzyme gave two bands on SDS-PAGE, which corresponded to molecular masses of 15 and 16 kDa (Fig. S2).

In order to identify the 7,8-DHF-converting enzyme, partial *N*-terminal amino acid sequencing was carried out. The 20 amino acid sequences of the two subunits were as follows: VLSAADKANVKAAWGIVGGQ (15 kDa small subunit) and VHLSAEEKEAVLGLWGKVNV (16 kDa large subunit). A search with the BLAST program revealed that these *N*-terminal amino acid sequences exhibited high similarity (95% and 100%, respectively) with those of hemoglobin (Hb) subunit alpha and beta of *Sus scrofa* (pig), respectively.

We then investigated whether the purified Hb is identical to blood Hb (from the same pig) or not. Blood Hb was purified from red blood cells of the pig with a HiPrep DEAE FF 16/10 column after hemolysis (Table S2). The purified blood Hb gave two bands on SDS-PAGE, and the apparent molecular mass of the blood Hb was the same as that of that from liver (Fig. S3). The molecular masses of the subunits of both proteins were also determined by MALDI-TOF/MS to be as follows: alpha subunit, 15065.991 (from liver) and 15065.619 (from blood); and beta subunit, 16064.434 (from liver) and 16064.710 (from blood). These molecular masses show that both subunits of Hb from liver are identical to the corresponding subunits of Hb from red blood cells. Furthermore, the molecular masses of the heme extracted from Hb of blood and liver were determined by LC-ESI-MS as described under “*Methods*”, both hemes exhibiting a *m*/*z* value of 616.2 in the positive ion mode. This *m*/*z* value corresponded with that of protoporphyrin IX and iron^16^. Therefore, the purified enzyme from porcine liver was identified as Hb, which is a tetrameric protein comprising two alpha subunits and two beta subunits (α_2_β_2_).

By using the purified Hb from liver, the product that shows the *m*/*z* value of 529.1631 was clearly formed (Fig. S4). Using CoA as a substrate, we next compared the activities of Hb from porcine blood and human blood with that of Hb from porcine liver (Table S3). Both of them exerted the activity, but it was lower than that of Hb from porcine liver.

### Pantetheine as a substrate for Hb

The results of mass spectrometry analysis described under “*Detection of a novel metabolite of 7,8-DHF*” indicated the reaction product was the pantetheine conjugate of 7,8-DHF. We thus tested pantetheine as a substrate for the Hb reaction. Pantetheine was prepared from pantethine (dimeric form of pantetheine) with a 4-fold higher concentration of DTT, and then purified by HPLC. The reaction was carried out in the standard assay mixture using purified pantetheine as a thiol substrate. In this reaction, the product exhibited the same *m*/*z* value and retention time ([M-H]^−^ ion at *m*/*z* 529.2 and 8.4 min) as the product observed when CoA was used as a substrate. On the other hand, the specific activity of Hb increased to 1.01 μmol/min/mg from 0.403 × 10^−3^ μmol/min/mg by using pantetheine as a substrate instead of CoA (Table 1).

**Table 1:** 7,8-DHF-converting activity of Hb from blood with various thiol donors.

Thiol donor	Specific activity (μmol/min/mg)
Glutathione	1.35 ± 0.12
Cysteine	1.29 ± 0.09
Pantetheine	1.01 ± 0.11
CoA	0.403 ± 0.033 (×10^−3^)

Hb from blood was added to the standard assay mixture. The final concentration of glutathione, cysteine, pantetheine, and CoA was 2 mM. Activities were calculated from the decrease in the amount of 7,8-DHF after reactions when glutathione, cysteine, and pantetheine were used as substrates.

### Structure determination of the pantetheine conjugate

**Figure 3:**
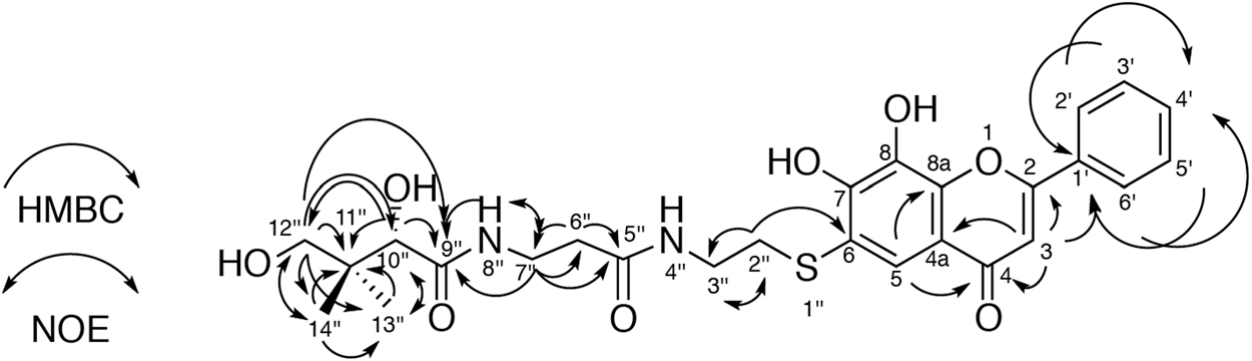
Determined structure of the pantetheine conjugate of 7,8-DHF.

The pantetheine conjugate of 7,8-DHF was prepared and purified as described under “*Methods*”. We determined its structure by NMR analysis (Fig. 3, Table S4 and Figs S5–9). Heteronuclear multiple bond coherence (HMBC) analysis revealed the correlation between H-2” of the pantetheine moiety and C-6 of the flavonoid moiety. This observation demonstrates that the thiol group of pantetheine was adducted to C-6 of 7,8-DHF.

### Degradation of CoA by Hb and Co-bound Hb

**Figure 4:**
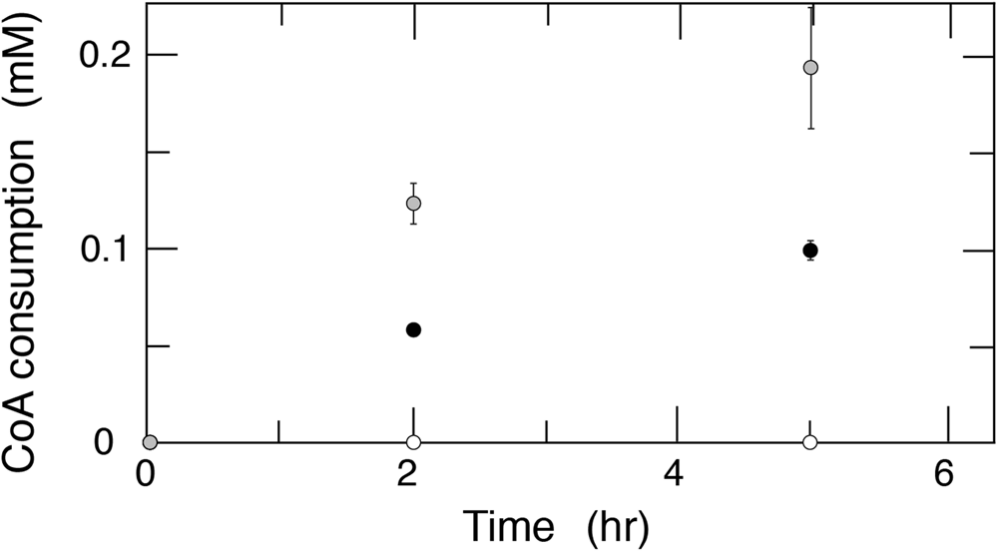
Degradation of CoA by Hb or CO-bound Hb. The standard reaction mixture containing 2.5 mg/mL Hb was incubated under air (gray) or CO (black). As a control, substrates were incubated under air (white). Amount of CoA was measured by LC/MS.

We then investigated the mechanism of degradation of CoA by Hb. When CoA was incubated with Hb, CoA was consumed and two products were observed (Fig. 4). The *m*/*z* values of the products were 506 [M-H]^−^ and 277 [M-H]^−^, indicating that Hb cleaved the CoA at the Z arrow in Fig. 2 to yield 3′-phospho-adenosine-5′-diphosphate (ADP) and pantetheine. In this reaction, the activity of CoA degradation by Hb was measured to be 0.657 ± 0.105 × 10^−3^ μmol/min/mg.

By LC/MS, we investigated this reaction by using CO treated Hb. The specific activity of CO-bound Hb was 0.311 ± 0.016 × 10^−3^ μmol/min/mg.

### Effect of the redox state of heme on the activity of Hb

We investigated the effects of the redox state and ligands on the activity of Hb. At first, we examined the effects of ligands (i.e., KCN and CO) and redox reagents (i.e., dithionite (Na_2_S_2_O_4_) and potassium ferricyanide (K_3_[Fe(CN)_6_]), under aerobic and anaerobic conditions. The spectra of the purified Hb drastically changed on the addition of various compounds (Fig. S10). These spectral shifts coincided with those reported previously^17^. The addition of CO or dithionite decreased the activity of Hb under anaerobic conditions. Also, the addition of potassium ferricyanide and KCN reduced the enzymatic activity by 60–80% under aerobic conditions (Table S5). These results showed that dioxygenated (Fe^2+^-O_2_) or oxidized (Fe^3+^) heme was required to convert 7,8-DHF to the corresponding conjugate.

### Effects of temperature and pH on the activity and stability of Hb

The effects of pH and temperature on the activity of Hb were examined (Fig. S11). The optimum pH and temperature were pH 8.5–9.0 and 45 °C, respectively. Hb was stable between pH 5.0 and pH 11.0, and under 50 °C.

The stability of Hb was examined at various pH values. After Hb had been incubated at 25 °C for 30 min in 100 mM buffers, citrate/sodium citrate buffer (pH 3.0–6.5), Hepes/NaOH buffer (pH 6.5–8.0), Tris/HCl buffer (pH 8.0–9.0), NH_4_Cl/NH_4_OH buffer (pH 9.0–10.0), and NaHCO_3_/NaOH buffer (pH 10.0–11.0), an aliquot of each enzyme solution was taken and assayed under the standard conditions. The activity of Hb was maintained even after high pH (pH 11.0) treatment. This is in agreement with previously reported results showing that the extent of denaturation of a hemolysate was relatively low after high pH (pH 12.5) treatment^18^.

The stability of Hb was examined at various temperatures. After Hb had been preincubated for 30 min in 100 mM Hepes-NaOH (pH 7.4), an aliquot of each solution was taken, and then the activity of Hb was assayed under the standard conditions. The activity of Hb was suddenly lost above 60 °C, which is in agreement with previously reported results showing that a rapid increase in the rate of denaturation of Hb was observed above 60 °C^19^.

### Kinetic properties of Hb

Using CoA and different concentrations of 7,8-DHF as substrates, a typical hyperbolic curve of pantetheine-conjugated product formation over substrate concentration was obtained, indicating that the reaction followed Michaelis-Menten-type kinetics (Fig. S12). The *K*_m_, *V*_max_, and *k*_cat_ values were 0.404 ± 0.081 mM, 0.466 × 10^−3^ ± 0.161 × 10^−3^ μmol/min/mg, and 1.24 × 10^−3^ s^−1^, respectively.

### Substrate specificity

**Figure 5:**
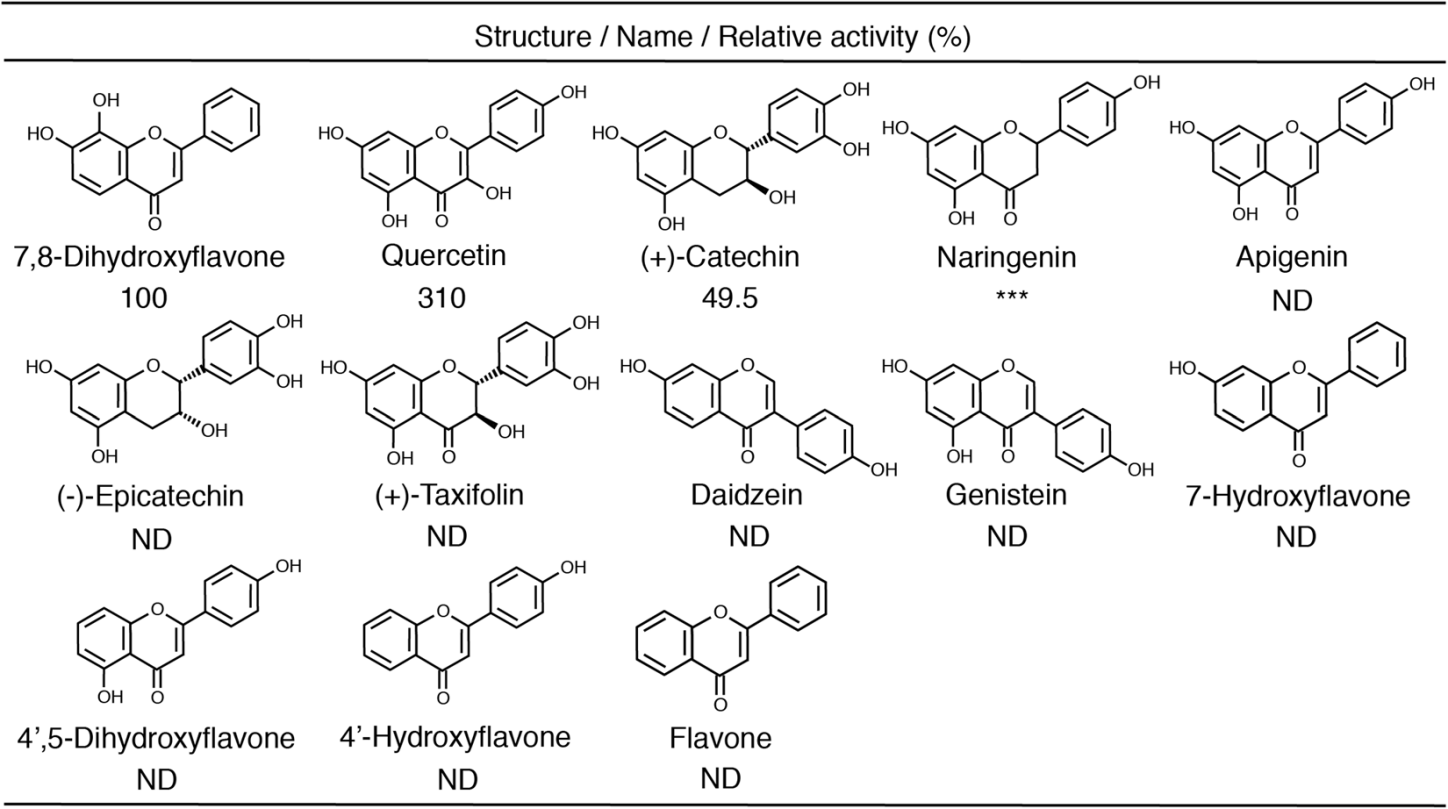
Substrate specificity of Hb. The reaction was carried out in the modified standard assay mixture; various flavonoids were used as substrates in place of 7,8-DHF at a concentration of 0.2 mM. ND, no product could be detected. ***The pantetheine conjugate was detected, but the peak intensity was too weak to calculate the specific activity. The experiments were carried out three times independently.

The substrate specificity of Hb toward various flavonoids was assayed by LC-ESI-MS in the standard assay mixture containing 2 mM CoA, 0.2–1 mM each flavonoid, and 1 mg/ml Hb. Among the tested flavonoids, quercetin, (+)-catechin, and naringenin were converted to the corresponding products, which exhibited the *m*/*z* values of pantetheine conjugates (Fig. 5). However, flavone, 7-hydroxyflavone (8-dehydroxylated derivative of 7,8-DHF), apigenin (3-dehydroxylated derivative of quercetin), (−)-epicatechin (diastereomer of (+)-catechin), 4′,5-dihydroxyflavone (3,7-dehydroxylated derivative of quercetin), and 4′-hydroxyflavone were inert as substrates for Hb. This indicates that the number and position of the hydroxyl group of a flavonoid is unrelated to the activity of Hb. On the other hand, Hb did not convert isoflavones (daidzein and genistein) into pantetheine conjugates. When pantetheine, glutathione, and cysteine were used as substrates instead of CoA, Hb was found to act on a much broader range of flavonoids including isoflavones (Table S6).

### Flavonoid-converting activity of heme-containing enzymes

We investigated the flavonoid-converting activity of the heme-containing enzymes, peroxidase and catalase. We incubated each of these enzymes with a thiol donor and 7,8-DHF and then analyzed the resultant reaction mixture by LC/MS. Both heme-containing enzymes showed flavonoid-converting activity when glutathione, cysteine or pantetheine were used as a thiol donor. While the conjugation-activity of Hb was higher than those of peroxidase and catalase when glutathione and cysteine were used as a substrate, that of catalase was higher than those of Hb and peroxidase when pantetheine was used as a substrate (Table 2).

**Table 2:** Flavonoid-converting activity of heme-containing protein.

Heme-containing protein	Specific activity (μmol/min/μmol heme)			
	Glutathione	Cysteine	Pantetheine	CoA
Hb (Porcine blood)	101 ± 13	99.1 ± 8.3	81.1 ± 5.4	0.033 ± 0.002
Peroxidase (Horse radish)	76.4 ± 8.6	54.6 ± 4.9	76.8 ± 3.8	N.D.
Catalase (Bovine liver)	9.77 ± 2.5	13.4 ± 1.8	154 ± 15	N.D.

The experiments were carried out three times independently.

### The effects of ROS scavengers on the activity of Hb

We detected non-enzymatic and time-dependent production of H_2_O_2_ in the presence of 7,8-DHF (Fig. S13), and the addition of H_2_O_2_ to the reaction mixture increased the activity 2.25-fold (Table S7). In a previous study, H_2_O_2_ was proposed to degrade Hb and to release the iron ion from Hb, which reacts with H_2_O_2_ to form reactive ^•^OH^20^. We then investigated the involvement of ROS in the 7,8-DHF-converting activity of Hb. We measured the 7,8-DHF-converting activity of Hb in the presence of various scavengers for such reactive species (i.e., H_2_O_2_, ^•^OH, O_2_^−^ and ^1^O_2_). In this experiment, the tested ROS scavengers increased the activity of Hb (Table S8).

## Discussion

In humans, flavonoids have several beneficial effects due to such as their antioxidant, anti-inflammatory, antitumor, and estrogenic activities. Because of these attractive functions, many flavonoids have been searched for and characterized in plants.

In animals, dietary flavonoids are absorbed from the intestine and converted into their conjugated metabolites in the epithelial cells by enzymes such as uridine-5′-diphosphoglucuronosyltransferase, phenolsulfotransferase, catechol-*O*-methyltransferase, and glutathione *S*-transferase. The resultant conjugated metabolites and each of the corresponding aglycones are excreted into the digestive tract or flow into the portal vein through specific transporter systems. Flavonoids absorbed from the intestine are transferred to and also metabolized in the liver. Then, some resulting metabolized flavonoids are transferred from the liver to the bloodstream, the others being mixed with bile and returned to the intestinal tract via the enterohepatic circulation^21^. Studies on the metabolic fate of flavonoids are important to understand the bioavailability and function of a flavonoid. However, there are flavonoids of which the metabolic pathways have not been clarified such as 7,8-dihydroxyflavone (7,8-DHF).

7,8-DHF exerts a protecting effect on neurons^22^, improves memory^23^, antagonizes depression^24^, and functions as a vasorelaxing and hypertensive agent^25^. Although these beneficial effects have attracted interest, the metabolism of 7.8-DHF has never been investigated.

In this study, we explored novel metabolism of a flavonoid in mammals, using pig liver and 7,8-DHF as the enzyme source and substrate, respectively. At last, we successfully identified CoA as another substrate for pantetheine-conjugating activity of cell-free extracts toward 7,8-DHF. Surprisingly, we found that Hb, which is usually considered as an oxygen transporter, catalyzed this conjugation reaction. We clarified that the novel Hb-catalyzed reaction consists of CoA degradation into pantetheine and 3′-phospho-ADP and synthesis of pantetheine conjugated flavonoids, which are novel compounds (Fig. 3).

As found in a previous study, the NH_2_ group of the Val-1(β), Lys-82(β) and Lys-144(β) of human Hb attacks the carbon of the ester bond (-COO-) of the methyl acetyl phosphate to yield acetylated Hb^26^. This is a type of chemical modification of Hb and such covalent chemical modification inhibits the sickling of red blood cells^27^. In another study, enzyme-like activities of Hb were found. For example, hydroxylase-^28^, hydrolase-^29^, oxygenase-, peroxidase- and catalase activities^30,31^ have been reported. Among these reactions, only the hydroxylation reaction was analyzed kinetically and it was shown to be a Michaelis-Menten-type reaction. In our study, the hydrolytic cleavage of CoA and conjugation of the resulting pantheteine to 7,8-DHF showed typical Michaelis-Menten kinetics, and *K*_m_ for 7.8-DHF was calculated to be 0.4 mM. To the best of our knowledge, conjugating activity of Hb toward flavonoids has never been reported. In Hb, the degradation of CoA into pantetheine would be a rate-limiting step, because the specific activities of Hb for pantetheine conjugation and that for CoA hydrolysis were 1.01 ± 0.11 μmol/min/mg and 0.657 ± 0.105 × 10^−3^ μmol/min/mg, respectively. In the common degradation pathway for CoA, CoA is first dephosphorylated (Fig. 2, arrow *X*), and then cleaved (Fig. 2, arrow *Y*) into 4′-phosphopantetheine and the resulting dephosphorylated adenosine moiety by CoA phosphatase and dephospho-CoA pyrophosphatase, respectively^32^. On the contrary, we found that Hb catalyzed the degradation of CoA at the cleavage site between the pantetheine moiety and phosphate (Fig. 2, arrow *Z*). This cleavage site is different from those for the CoA-degrading reactions catalyzed by the above enzymes. Moreover, carbon monoxide (CO) did not inhibit this reaction catalyzed by Hb, demonstrating that the hydrolysis of CoA was carried out independent of heme. This is the first example that Hb showed the enzymatic activity regardless of the heme.

In general, heme functions as an active site in heme-containing enzymes. Because Hb has a heme in each subunit, we assumed that heme is involved in the catalysis of the 7,8-DHF-converting reaction. To examine this speculation, we investigated the effects of redox reagents and exogenous ligands on the 7,8-DHF-converting activity of Hb. CO is known to bind to the ferrous iron (Fe^2+^) of Hb with much higher affinity than O_2_, i.e., about 210-fold higher^33^. On the other hand, potassium cyanide (KCN) specifically binds to the ferric iron (Fe^3+^). Enzyme assays under these conditions have revealed that reduced and dioxygenated (Fe^2+^-O_2_) or oxidized (Fe^3+^) heme was required for the 7,8-DHF-converting activity of Hb with CoA as a substrate. In the blood circulatory system, Hb with Fe^2+^-O_2_ and Fe^3+^ accounts for a large portion of total Hb. This suggests that Hb could exert flavonoid-converting activity under physiological conditions. In previous studies, Hb was found to catalyze hydroxylation and peroxidation of aniline^29^ and anthracene^31^, respectively. Both reactions are suggested to be catalyzed by heme in Hb. This means the pocket around heme is enough wide for a flavonoid (whose size is similar to the above aromatic compounds) to bind. Therefore, the reaction of pantetheine conjugation to 7,8-DHF possibly proceeds on the heme-binding site. However, it is not clear that the space around the heme of Hb is wide enough for binding of CoA, because CoA is bigger than 7,8-DHF. We assume that the hydrolysis of CoA would proceed on a site other than the heme-binding site of Hb. In heme-containing enzymes, reactions in which hydrogen peroxide acts as a mediator are also known^34^. In our study, however, reactive oxygen species (ROS) scavenger experiments showed that ROS including hydrogen peroxide are not involved in this reaction.

We here propose a possible reaction mechanism of pantetheine conjugation after CoA hydrolysis by analogy with the hydroxylation catalyzed by cytochrome P450s. Cytochrome P450s catalyze the hydroxylation of substrates after the formation of the key intermediate Compound I (Fe^4+•^=O). The heme iron of cytochrome P450 binds to O_2_, and then forms Compound I via electron transfer from NAD(P)H to the oxygen atom in the P450-catalyzed reaction. We speculate that Hb catalyzes the pantetheine conjugation of a flavonoid via the formation of the same intermediate Compound I using pantetheine as an electron donor instead of NAD(P)H. A possible reaction mechanism of the pantetheine conjugation catalyzed by Hb is as follows: (*i*) the oxygen of oxygenated ferrous heme (Fe^2+^-O_2_) of Hb is reduced by pantetheine (in the case of ferric heme [Fe^3+^], it is reduced to ferrous [Fe^2+^] by thiol, and then the resulting Fe^2+^ binds to O_2_ before the reduction by pantetheine described above), (*ii*) through protonation of the resulting reduced oxygen (Fe^2+^-O-O^−^), the intra-molecular O-O bond is cleaved to yield Compound I (Fe^4+•^=O) with heme, (*iii*) Compound I abstracts the proton of C-6 of 7,8-DHF, and (*iv*) the resulting electrophilic C-6 is attacked by thiol to form a thiol conjugate. Induction of the 7,8-DHF-converting activity (2.25-fold) of Hb by H_2_O_2_ (Table S7) would support this hypothesis, because it is proposed that H_2_O_2_ reacts with Hb and forms a compound I-like ferryl derivative (Fe^4+^=O) with a radical site on a porphyrin or protein^35^. Furthermore, peroxidase and catalase may also catalyze pantetheine conjugation of 7,8-DHF in similar mechanisms, as Compound I and mechanisms of its formation involving dioxygen or its derivatives are likely to be a shared feature across those heme-proteins^36^.

Interestingly, Hb was also able to conjugate flavonoids with glutathione as well as pantetheine (Table 1, Table S6). Although glutathione conjugation is an important metabolic reaction, in general, glutathione *S*-transferase is the only known enzyme that catalyzes this reaction^37^. Expression of glutathione *S*-transferase is induced by various compounds, such as aromatic compounds or isothiocyanates, that are recognized as substrates by this enzyme. Flavonoids also induce the expression of glutathione *S*-transferase^38^, but flavonoids cannot act as substrates for glutathione *S*-transferase. It is proposed that flavonoids are inhibitors rather than substrates for glutathione *S*-transferase, because flavonoids bind to the cysteine residue near the active site of this enzyme^39^. Although a glutathione conjugate of quercetin has been detected and identified on LC-MS/MS in humans^40^, glutathione *S*-transferase has not been identified as the responsible enzyme because of this inhibitory effect of flavonoids. In the present study, we found that Hb had glutathione-conjugating activity. Taking into account the abundance of Hb in the liver, Hb may act as an alternative metabolic enzyme for glutathione conjugation of flavonoids after absorption in the small intestine. In other words, Hb could make up for the lack of the ability of glutathione *S*-transferase as to flavonoids.

We also found that Hb can act on other flavonoids as well as 7,8-DHF (Fig. 5, Table S6). The finding that Hb can act on these flavonoids (quercetin, catechin and naringenin) shows that a wide range of flavonoids in dietary plants are recognized as substrates by Hb. Furthermore, this is the first report showing that isoflavones, whose glutathione or cysteine conjugates have never previously been reported, undergo pantetheine conjugation catalyzed by Hb. In general, glutathione conjugation of a variety of polyphenols frequently enhances redox activity, and the conjugates exhibit a wide array of cellular activities^41^. As pantetheine conjugation of flavonoids has never been reported before, elucidation of the bioactivity and metabolic fate of the conjugates is expected.

Hb is the most studied protein in human history, in terms of physiological function and molecular structure. Although the primary role of Hb in vertebrates is to transport molecular oxygen, our findings are the first to show that Hb can act as an enzyme that converts flavonoids into the thiol conjugates using CoA, pantetheine, glutathione, or cysteine as a substrate. Human Hb was also found to have 7,8-DHF-converting activity (Table S3). We thus suggest that the novel activity is common to Hb of both pigs and humans.

A recent hypothesis^42^ proposes that an oxygen-transport protein, hemocyanin, is an origin of tyrosinase and catechol oxidase which catalyze oxidative reactions and share the distinct metal-containing structure of the active site with hemocyanin. However, the fact that hemocyanin also catalyzes tyrosinase- and catechol oxidase-like phenol oxidation indicates that the oxygen-transport protein can catalyze the oxidative reaction according to the substrate-binding geometry^43^. Therefore it is not unreasonable that Hb, which is also a metal-containing oxygen transport protein, oxidatively added each of the thiol substrates to 7,8-DHF in our study. On the other hand, it is very surprising that Hb catalyzes the hydrolysis of CoA into pantetheine and 3′-phospho-ADP independently of heme of Hb. This hydrolytic activity is a new function of Hb.

Whether the substrates used in this study are the physiological substrates for Hb or not, our discovery of the novel ability of Hb is important. Although the specific activity of the Hb-catalyzed reaction is low, this does not rule out the possibility that the enzymatic activity of Hb, which exists abundantly in liver and blood, is physiologically significant. As physiological importance of thiol conjugates of flavonoids was not investigated, elucidation of their effects on human is the next challenge. Our findings pave the way to further studies on this new function, in which heme or polypeptide regions in Hb are involved, and new physiological roles are revealed for this ubiquitous protein.

## Methods

Matrix-assisted laser desorption ionization time-of-flight mass spectrometry (MALDI-TOF/MS) analysis, Quantification of the reaction product, Structural analysis of the reaction product, Quantification of H_2_O_2_ production in the presence of 7,8-DHF, Effects of various ROS scavengers on the 7,8-DHF-converting activity of Hb.

Please see Supplementary Methods.

### Materials

7,8-Dihdroxyflavone, 7-hydroxyflavone, 4′-hydroxyflavone, flavone, quercetin, naringenin, and 4-aminoantipyrine were purchased from Tokyo Kasei Kogyo Co., Ltd. (Tokyo, Japan). (+)-Catechin, pantethine, superoxide dismutase (from bovine liver), hemoglobin (from humans), and carboxypeptidase B were purchased from Sigma (Missouri, USA). 4′,5-Dihydroxyflavone was purchased from Alfa Aesar (Massachusetts, USA). CoA and peroxidase (from horseradish) were purchased from Oriental Yeast (Tokyo, Japan). Catalase (from bovine liver) was purchased from Wako Pure Chemical Industries, Ltd. (Osaka, Japan). Resource Q, HiPrep DEAE FF 16/10, and a low molecular weight standard kit were obtained from GE Healthcare (Buckinghamshire, UK). Bio-Scale mini cartridges CHT Type I 40 µm media (5 ml) were obtained from Bio-Rad (California, USA). The “Protease inhibitor cocktail for use with mammalian cell and tissue extracts” was purchased from Nacalai Tesque (Kyoto, Japan). *N*-Ethyl-*N*-(2-hydroxy-3-sulfopropyl)-3,5-dimethoxyaniline was purchased from Dojindo (Kumamoto, Japan). All other chemicals used were from commercial sources and of analytical grade. Porcine liver and blood were collected from the same individual.

### Analysis of the reaction product obtained on incubation with cell-free extracts

Porcine liver was homogenized using a POLYTRON PT 10–35 GT (Kinematica, Lucerne, Switzerland), and then the cells were disrupted by sonication (Insonator model 201M; Kubota, Tokyo, Japan) in phosphate-buffered saline (PBS) containing 1% (vol/vol) of protease inhibitors (as described under “*Materials*”). Cell debris was removed by centrifugation (12,000 *g* × 20 min). The resulting supernatant was used as a cell-free extract. The reaction mixture comprised 50 mM Hepes-NaOH buffer (pH 7.4), 5 mg/ml protein, 1 mM 7,8-DHF (in dimethyl sulfoxide [DMSO]), and 1 mM cofactor in a total volume of 200 µl. The reaction was started by adding a cell-free extract, followed by incubation for 10 h at 37 °C. The reaction product was analyzed by liquid-chromatography electrospray-ionization mass spectrometry (LC-ESI-MS) and high-resolution mass spectrometry (HRMS). LC-ESI-MS analysis was performed with a Nexera X2 system (Shimadzu, Kyoto, Japan) equipped with a Cosmosil πNAP column 4.6 × 150 mm (Nacalai Tesque). HRMS analysis was performed with a UPLC/Synapt G2 HDMS (Waters, Massachusetts, USA) equipped with an ACQUITY UPLC BEH C18 column (Waters).

### Purification of the 7,8-DHF-converting enzyme from porcine liver

All purification procedures were performed at 0–4 °C. Cell-free extracts were prepared as described above and fractioned with ammonium sulfate (40–60% saturation), followed by dialysis against 20 mM Tris-HCl buffer (pH 8.0). Each dialyzed solution was applied to a Resource Q column (6 ml) equilibrated with the same buffer. Protein was eluted from the column by increasing NaCl linearly from 0 to 1 M in the same buffer. The active fractions were collected and then dialyzed against 1 mM potassium phosphate buffer (pH 7.5). The protein solution was applied to a CHT Type I column equilibrated with the same buffer. The protein was eluted by increasing the concentration of the potassium phosphate buffer linearly from 0 to 500 mM. The active fractions were collected and then dialyzed against 20 mM Tris-HCl buffer (pH 8.0). The protein solution was applied to a Resource Q column (6 ml) equilibrated with the same buffer. Protein was eluted from the column by increasing NaCl linearly from 0 to 1 M. The active fractions were collected and then dialyzed against 10 mM Hepes-NaOH buffer (pH 7.4). The homogeneity of the purified protein was confirmed by SDS-PAGE. The relative molecular mass of the enzyme subunit was determined from the relative mobility of marker proteins on SDS-PAGE, phosphorylase *b* (97 kDa), BSA (66 kDa), ovalbumin (45 kDa), carbonic anhydrase (30 kDa), soybean trypsin inhibitor (20.1 kDa), and alpha-lactalbumin (14.4 kDa).

### Enzyme assays

All of the reactions were performed under linear conditions with an appropriate amount of protein and a suitable reaction time, unless otherwise noted. The standard assay mixture comprised 100 mM Hepes-NaOH buffer (pH 7.4), 1 mM 7,8-DHF (in DMSO), 2 mM CoA, and an appropriate amount of enzyme in a total volume of 200 µl. This standard assay mixture was used for the assaying of 7,8-DHF-converting activity, with the exception of investigation of the substrate specificity, for which the concentration of flavonoids was 0.2 mM. In these standard assays, the reaction was started by addition of the enzyme and carried out at 37 °C. The reaction was stopped by the addition of 200 µl acetonitrile to the reaction mixture, and a supernatant was obtained by centrifugation (20,630 × *g*, 10 min).

One unit of the 7,8-DHF-converting activity was defined as the amount of the enzyme that catalyzed the production of 1 µmol pantetheine conjugate per hour under the standard assay conditions. The concentration of the pantetheine conjugate was calculated from a standard curve established from the peak area of the purified pantetheine conjugate of 7,8-DHF in a mass chromatogram (*m*/*z* 529.2 [M-H]^−^). Specific activity is expressed as units per milligram of protein. The protein concentrations were determined with a protein assay kit (Nacalai Tesque) using bovine serum albumin as the standard, as in the method of Bradford^44^.

### Flavonoid-converting activity of heme-containing enzymes

Heme contents of heme-containing enzymes were analyzed with a pyridine hemochrome method^45^. An extinction coefficient value for heme B pyridine hemochrome of 34.4 mM^−1^ cm^−1^ at 557 nm was used^46^. Each heme-containing enzyme was added to the standard assay mixture. The final concentration of each heme-containing enzyme was 25 µM. After incubation at 37 °C, reaction mixtures were analyzed by LC/MS.

### Substrate specificity

The following flavonoids were examined as to substrate specificity at final concentrations of 0.2–1 mM: quercetin, genistein, daidzein, apigenin, naringenin, (+)-catechin, (−)-epicatechin, (+)-taxifolin, 7-hydroxyflavone, 4′, 5-dihydroxyflavone, 4′-hydroxyflavone, and flavone. Each of these flavonoids was added to standard assay mixture instead of 7,8-DHF. The production of the pantetheine conjugates was detected by LC-ESI-MS.

## Electronic supplementary material

Supporting information
