# Supplementary material for: Hemoglobin catalyzes CoA degradation and thiol addition to flavonoids
Source: Sci Rep. 2018 Jan 19;8:1282. doi: 10.1038/s41598-018-19585-7 (PMC5775311; doi:10.1038/s41598-018-19585-7)
Supplement: Supplementary file 1 — Supporting information [file 41598_2018_19585_MOESM1_ESM.pdf]

Supporting Information

Hemoglobin catalyzes CoA degradation and thiol addition to flavonoids

Toshiki Nagakubo[a,1], Takuto Kumano[a,1], Yoshiteru Hashimoto[a] and Michihiko Kobayashi[a,2]

[a] Graduate School of Life and Environmental Sciences, University of Tsukuba,

1-1-1 Tennodai, Tsukuba, Ibaraki 305-8572, Japan.

[1] T. N. and T. K. contributed equally to this work.

[2] To whom correspondence should be addressed.

Contents:

Methods, Tables S1-S8 and Figures S1-S13

Supplementary Methods

Matrix-assisted laser desorption ionization time-of-flight mass spectrometry (MALDI-TOF/MS) analysis

MALDI-TOF/MS was performed using an Ultraflextreme-ETA MALDI-TOF/TOF (Bruker) in the linear mode. One μl of a 3 mg/ml Hb solution was deposited onto the target position on a sample plate and allowed to dry. One μl of a matrix solution (saturated solution of sinapic acid in acetonitrile with 0.1% trifluoroacetic acid) was then deposited onto the sample spot and allowed to dry before insertion into the MALDI-TOF mass spectrometer.

Purification of Hb from porcine blood

Hb was purified from porcine blood as follows. Erythrocytes were obtained by centrifugation (1,000 g × 10 min), and then washed twice with PBS (phosphate-buffered saline). A 3-fold excess of deionized water was then added. Cell debris was removed by centrifugation. After dialysis against 20 mM Tris-HCl buffer (pH 8.0), the protein solution was applied to a HiPrep DEAE FF 16/10 column equilibrated with the same buffer. Protein was eluted from the column by increasing NaCl linearly from 0 to 1 M. The fractions containing Hb were selected as to absorption at 415 nm and then dialyzed against 10 mM Hepes-NaOH (pH 7.4). The homogeneity of the purified Hb was confirmed by SDS-PAGE.

Extraction and mass spectrometry analysis of heme

Heme was extracted from Hb by Teale's 2-butanone method[46]. One hundred μl of a 2 mg/ml Hb solution was acidified to pH 2.5 using 0.1 M HCl and the heme was extracted by the addition of ice cold 2-butanone. The extract was evaporated and the residue was dissolved in 50 μl of methanol. The separation and MS analysis of the extract were performed by LC-ESI-MS with a Shimadzu Nexera X2 system equipped with a TSK-gel ODS-100V column, 4.6 × 150 mm (Tosoh Co., Tokyo, Japan), under the following conditions: column temperature, 40ºC; gradient elution; mobile-phase solvent A [0.05% (vol/vol) formic acid in deionized H_2O] and solvent B [CH_3CN]; mobile-phase composition, 70% solvent A/30% solvent B at 0 min, 20% solvent A/80% solvent B at 10 min, and 70% solvent A/30% solvent B at

15 min; and flow rate, 1.0 mL/min. The MS analysis data were acquired with a mass spectrometer with ESI in the positive and negative modes.

Quantification of the reaction product

The amount of the product was determined by LC-ESI-MS under the following conditions: column temperature, 40˚C; gradient elution; mobile-phase solvent A [0.05% (vol/vol) formic acid in deionized H_2O] and solvent B [CH_3CN]; mobile-phase composition, 100% solvent A at 0 min, 30% solvent A/70% solvent B at 10.5 min, and 100% solvent A at 11.5 min; flow rate, 1.0 mL/min; and photodiode array detector, 190~600 nm. The MS analysis data were obtained with a mass spectrometer with ESI in the positive and negative modes.

Structural analysis of the reaction product

For structural analysis of the reaction product, a preparative-scale reaction was carried out through the following procedures. The reaction mixture consisted of 50 mM Hepes-NaOH, 0.3 mg/ml purified Hb, 1 mM 7,8-DHF, 1 mM pantethine, and 4 mM dithiothreitol in a total volume of 300 ml. The reaction mixture was incubated at 37ºC for 20 h, and then extracted with 300 ml of ethyl acetate and 3 ml of acetate. The extract was evaporated and the residue was dissolved in 2 ml of methanol. The pantetheine conjugate was purified by high-performance liquid chromatography (HPLC) with a Shimadzu LC-10Avp system equipped with a Cosmosil πNAP 20 × 150 mm column (Nacalai Tesque) under the following conditions: column temperature, 40ºC; isocratic elution; mobile-phase composition, 40% deionized water/60% methanol (vol/vol); flow rate, 7 ml/min; and detection at 274 nm. The pantetheine conjugate was evaporated and the residue was dissolved in DMSO-d_6. The chemical structure of the pantetheine conjugate was elucidated by ^{1}H NMR, ^{13}C NMR, HMBC (heteronuclear multiple bond coherence), and NOE (nuclear overhauser effect) analyses using an AVANCE-600 NMR spectrometer (Bruker, Massachusetts, USA).

Quantification of H_2O_2 production in the presence of 7,8-DHF

The reaction mixture comprising 50 mM Hepes-NaOH (pH 7.4), 1 mM 7,8-DHF, 1 mM *N*-ethyl-*N*-(2-hydroxy-3-sulfopropyl)-3,5-dimethoxyaniline, 1 mM 4-aminoantipyrine, and 50 U/ml

horseradish peroxidase was incubated at 37°C. H_2O_2 was quantified using the absorbance and extinction coefficient of blue pigment derived from *N*-ethyl-*N*-(2-hydroxy-3-sulfopropyl)-3,5-dimethoxyaniline and 4-aminoantipyrine at 595 nm.

Effects of various compounds on the 7,8-DHF-converting activity of Hb

Various compounds were investigated as to their effects on the 7,8-DHF-converting activity of Hb. Each metal and inhibitor was added to the standard assay mixture to the final concentrations of 5 and 2 mM, respectively. The final concentration of Hb was 1 mg/mL.

Effects of various ROS scavengers on the 7,8-DHF-converting activity of Hb

Various ROS scavengers were added to the standard assay mixture. The final concentration of each ROS scavenger was as follows: superoxide dismutase, 0.1 mg/ml; peroxidase, 0.1 mg/ml; catalase, 0.1 mg/ml; riboflavin, 0.1 mM; mannitol, 5 mM; salicylate, 5 mM; urea, 5 mM; and phenylalanine, 5 mM.

Supplementary Results

Effects of various compounds on the activity of Hb

The effects of various compounds on the activity of Hb were investigated (Table S7). $MgCl_2$ was found to increase the activity of Hb, probably due to the promotion of hydrolysis of phosphate esters by divalent cations. Hb was sensitive to $CdCl_2$, $FeCl_3$, $FeCl_2$, $AlCl_3$, $HgCl_2$, $MnCl_2$, $CoCl_2$, $NiCl_2$, $CuCl_2$, $SrCl_4$, and $AgNO_3$. Phenylhydrazine and hydroxylamine inhibited the activity of Hb, indicating the binding of these reagents to heme iron. Inhibition by SH-reagents could be attributed to reactivity to the SH group of CoA. One chelating agent, diethyldithiocarbamate, caused strong inhibition, while the others exerted moderate or no inhibition. Reducing agents, dithiothreitol and 2-mercaptoethanol, also inhibited the activity, probably in a similar manner to in the case of dithionite (Fig. S10 and Table S5). As for oxidizing agents, ammonium persulfate and H_2O_2 showed contradictory effects on the activity of Hb. Serine modifiers partially inhibited the activity of Hb (phenylmethanesulfonyl fluoride, 51.3%; diisopropyl fluorophosphate, 63.7%).

SI Tables

Table S1. Purification of the 7,8-DHF converting enzyme.

Step	Total protein (mg)	Total activity (units×10^3)	Specific activity (units/mg×10^3)	Yield (%)
Cell-free extract	6200	718	0.116	100
$(NH_4)_2SO_4$	236	115	0.487	16.0
Resource Q	61.6	11.6	0.189	1.62
CHT Type I	5.30	4.70	0.886	0.655
Resource Q	1.86	1.65	0.885	0.230

Table S2. Purification of Hb from porcine blood.

Step	Total protein (mg)	Total activity (μmol/min×10^3)	Specific activity (μmol/min/mg×10^3)	Yield (%)
Hemolysate	1140	141	0.124	100
HiPrep DEAE FF 16/10	123	21.8	0.177	15.5

Table S3. 7,8-DHF-converting activity of Hbs from various sources.

The specific activities of Hb from different sources when CoA was used as the substrate were compared as relative activity. Experiments were carried out three times independently.

Source	Relative activity (%)
Porcine liver	100±2
Porcine blood	48.1±2.5
Human blood	25.2±0.8

Table S4. NMR spectra data for the pantetheine conjugate of 7,8-DHF.

No.	^{1}H		J_H (Hz)		^{13}C
1					
2					161.5
3	6.80	s		1H	106.3
4					176.5
4a					114.4
5	7.36	s		1H	115.1
6					121.8
7					165.2
8					134.1
8a					145.3
1'					132.3
2'	8.12	m		1H	126.7
3'	7.57	m		1H	129.4
4'	7.57	m		1H	131.7
5'	7.57	m		1H	129.4
6'	8.12	m		1H	126.7
1"					
2"	2.94	dd	7.6	2H	31.9
3"	3.26	dd	11.5, 6.8	2H	38.6
4"	8.43	br		1H	
5"					171.0
6"	2.30	dd	7.5	2H	35.6
7"	3.33	ddd	13.3, 7.0, 6.3	2H	35.3
8"	7.74	dd	5.6	1H	
9"					173.3
10"	3.71	s		1H	75.5
11"					39.5
12"	3.17, 3.30	d	10.3	2H	68.5
13"	0.79			3H	20.8
14"	0.81			3H	21.4

Table S5. Effect of the redox state of heme on the activity of Hb.

The final concentrations of $Na_2S_2O_4$ and KCN were 2 and 10 mM, respectively. $K_3[Fe(CN)_6]$ was added to the Hb solution to a final concentration of 2 mM before starting the reaction, and then removed by ultrafiltration to prevent the formation of S-S bonds between CoAs. The experiments were carried out three times independently. ND, not detected.

Condition	Redox Reagent	Exogenous ligand	Relative activity (%)
Aerobic			100±3
Aerobic	$K_3[Fe(CN)_6]$		140±17
Aerobic	$K_3[Fe(CN)_6]$	KCN	29.1±2.4
Aerobic		KCN	41.1±0.5
Anaerobic			83.6±0.3
Anaerobic	$Na_2S_2O_4$		N.D.
Anaerobic	$Na_2S_2O_4$	CO	N.D.
Anaerobic		CO	N.D.

Table S6. Substrate specificity of Hb when thiols were used as substrates.

The 7,8-DHF-converting activities of Hb were compared using various flavonoids and thiols. Activities were calculated from the decrease in the amount of each flavonoid after the reaction. ND, no product could be detected. ***, The conjugate was detected, but the peak intensity was too weak to calculate the specific activity. Experiments were carried out three times independently.

Flavonoid	Specific activity (μmol/min/mg)		
	Pantetheine	Glutathione	Cysteine
7,8-DHF	0.943	0.420	0.442
Quercetin	2.51	0.609	2.89
Apigenin	1.10	***	N.D.
7-Hydroxyflavone	***	N.D.	N.D.
4',5-Dihydroxyflavone	N.D.	N.D.	N.D.
4'-Hydroxyflavone	N.D.	N.D.	N.D.
Flavone	N.D.	N.D.	N.D.
Naringenin	1.67	***	N.D.
(+)-Catechin	2.06	0.0938	0.303
(-)-Epicatechin	2.04	0.280	0.684
(+)-Taxifolin	0.721	***	0.101
Daidzein	***	N.D.	N.D.
Genistein	***	N.D.	N.D.

Table S7. Effects of various compounds on the 7,8-DHF-converting activity of Hb.

Each compound was added to the standard reaction mixture, and assaying of Hb was performed after adding the substrate. The final concentrations of the tested compounds were as follows; metal salts, 5 mM; other inhibitors, 2 mM, unless otherwise stated. Experiments were carried out three times independently. ND, not detected.

Inhibitor	Relative activity (%)
None	100±3
$MgCl_2$	162±1
NaCl	120±1
LiCl	118±4
$RbCl_2$	118±1
$CaCl_2$	113±2
KCl	90.9±2.7
$ZnCl_2$	63.4±2.2
$CdCl_2$	59.6±4.6
$AlCl_3$	41.9±1.3
$FeCl_2$	40.7±1.1
$FeCl_2$	35.0±0.6
$HgCl_2$	25.7±0.4
$MnCl_2$	N.D.
$CoCl_2$	N.D.
$NiCl_2$	N.D.
$CuCl_2$	N.D.
$SrCl_4$	N.D.
$AgNO_3$	N.D.

Continued on next page

Table S7. (Continued from the previous page)

Inhibitor	Relative activity (%)
5,5'-Dithiobis-2-nitrobenzoate	12.1±1.2
Iodoacetate	56.3±3.3
N-Ethylmaleimide	2.56±0.81
p-Chloromercuribenzoate	3.66±0.48
Hydroxylamine	19.9±1.6
Phenylhydrazine	N.D.
Semicarbazide	110±9
Aminoguanidine	125±3
α, α-Dipyridyl	92.5±12.5
o-Phenanthroline	90.1±5.9
8-Hydroxyquinoline	99.0±6.1
EDTA	147±3
Diethyldithiocarbamate	7.26±0.60
NaN_3	99.2±5.9
Dithiothreitol	97.3±2.9
Dithiothreitol (10 mM)	26.5±1.9
Dithiothreitol (20 mM)	N.D.
2-Mercaptoethanol	21.0±4.7
H_2O_2	225±20
Ammonium persulfate	42.5±3.6
Phenylmethanesulfonyl fluoride	51.3±2.9
Diisopropyl fluorophosphate	63.7±0.3

Table S8. Effects of various ROS scavengers on the Hb activity.

Each of various ROS scavengers was added to the standard assay mixture. The specific activity of Hb under each condition was compared as relative activity. Experiments were carried out three times independently.

Scavenger	Relative activity (%)
None	100±7.87
Superoxide dismutase	99.3±1.36
Peroxidase	109±5.59
Catalase	102±1.10
Riboflavin	191±7.83
Mannitol	112±1.54
Salicylate	108±1.25
Urea	121±11.0
Phenylalanine	107±8.82

SI Figures

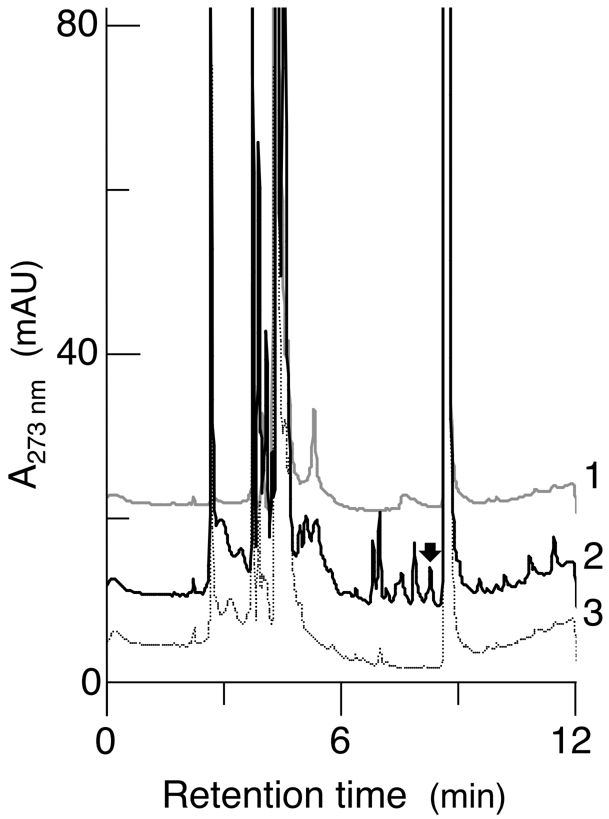

Figure S1. 7,8-DHF-converting activity of porcine liver.

A 7,8-DHF-derived reaction product was produced by cell-free extract of porcine liver in the presence of CoA. *1*, 7,8-DHF and CoA were incubated without the cell-free extract. *2*, 7,8-DHF and CoA were incubated with the cell-free extract. *3*, 7,8-DHF, CoA and the cell-free extract were mixed and then analyzed without incubation. All incubations were carried out at 37°C for 7 hours. Reaction mixtures were analyzed by LC/MS. The arrow denotes the peak of the 7,8-DHF-derived reaction product exhibiting a *m/z* value of 529.2 in the negative ion mode.

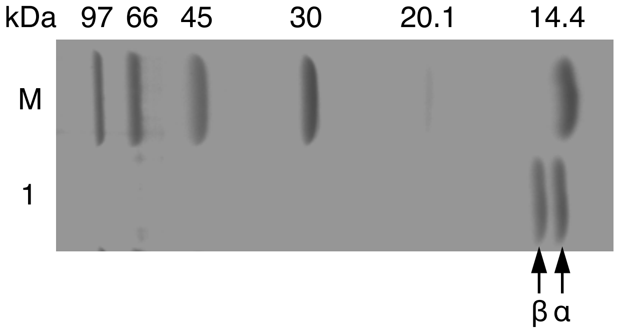

Figure S2. Purification of Hb.

SDS-PAGE of the purified Hb. Protein bands were detected by staining with Coomassie brilliant blue R-250. Lane M, marker proteins; lane 1, purified Hb. The alpha subunit and beta subunit are indicated by "α" and "β", respectively.

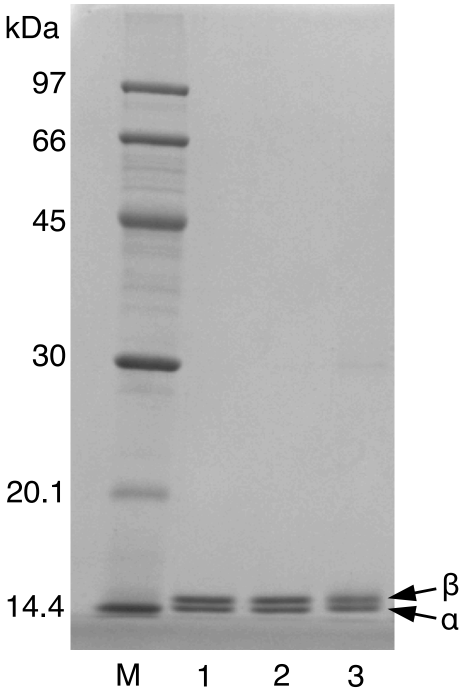

Figure S3. SDS-PAGE of Hb from porcine liver, porcine blood, and human blood.

Lane 1, Hb from porcine liver; lane 2, Hb from porcine blood; lane 3, Hb from human blood. The alpha subunit and beta subunit are indicated by "α" and "β", respectively.

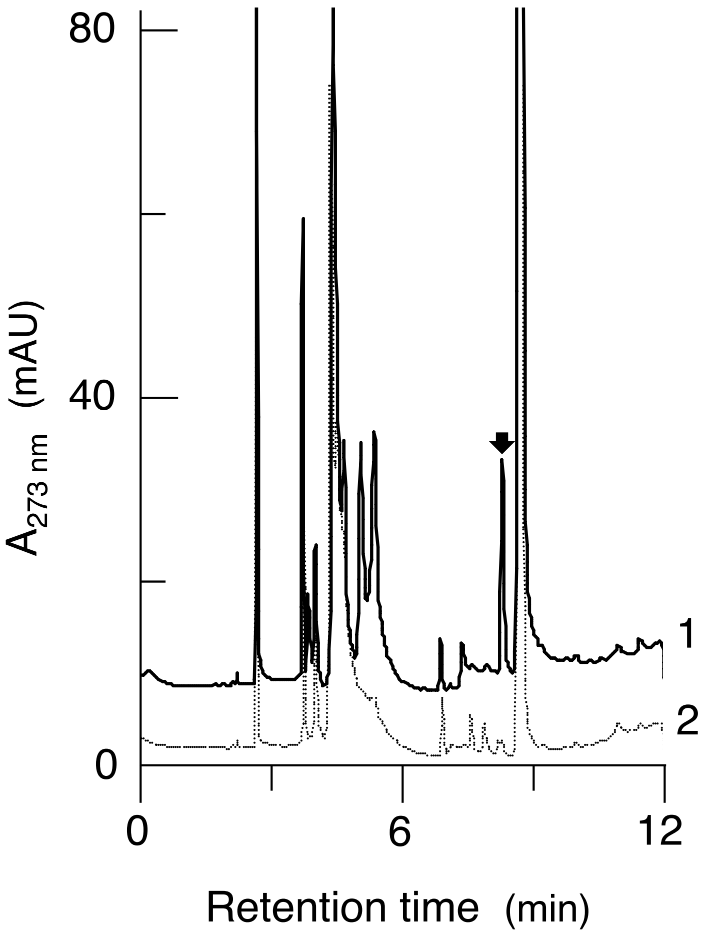

Figure S4. 7,8-DHF-converting activity of Hb from porcine liver.

A 7,8-DHF-derived reaction product was produced by Hb in the presence of CoA. 1, 7,8-DHF and CoA were incubated with Hb. 2, 7,8-DHF, CoA and Hb were mixed and then analyzed without incubation. All incubations were carried out at 37 °C for 7 hours. Reaction mixtures were analyzed by LC/MS. The arrow denotes the peak of the 7,8-DHF-derived reaction product exhibiting a m/z value of 529.2 in the negative ion mode.

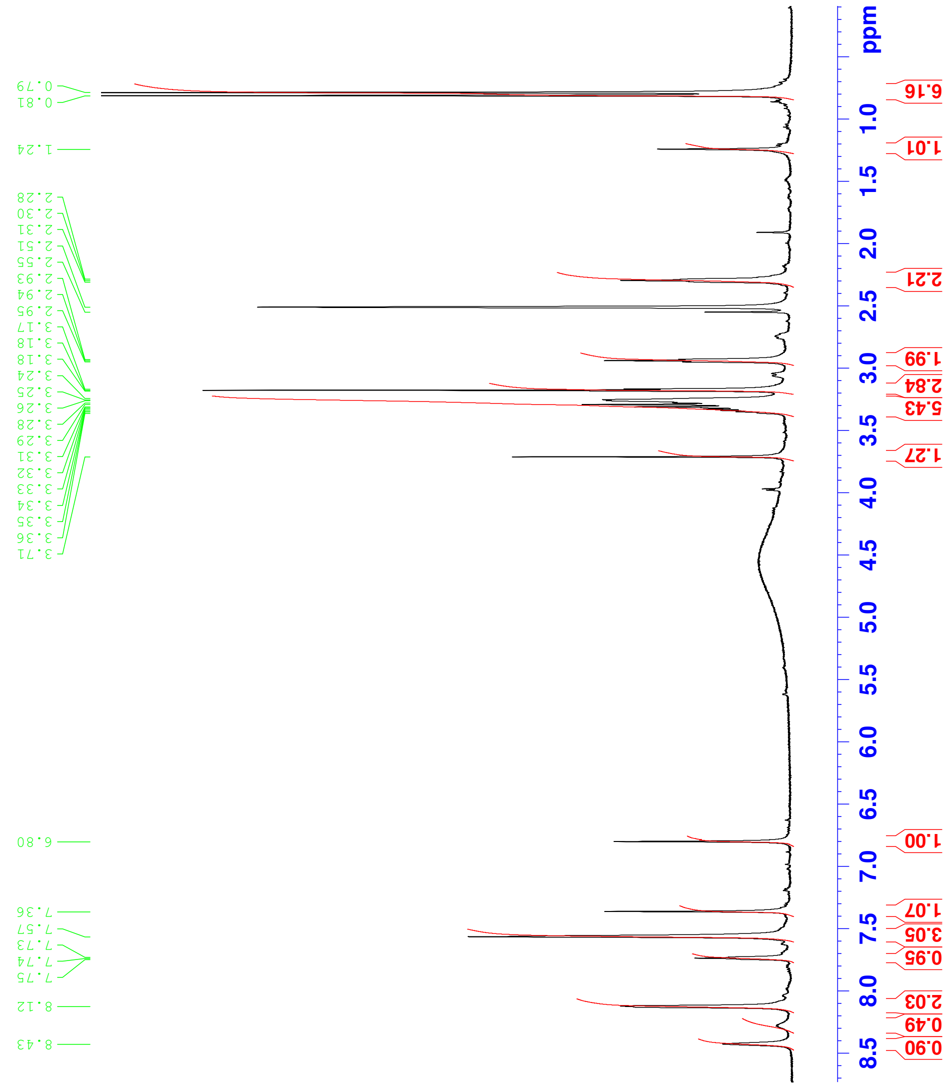

Figure S5. 1H spectrum of the pantetheine-conjugated 7,8-DHF.

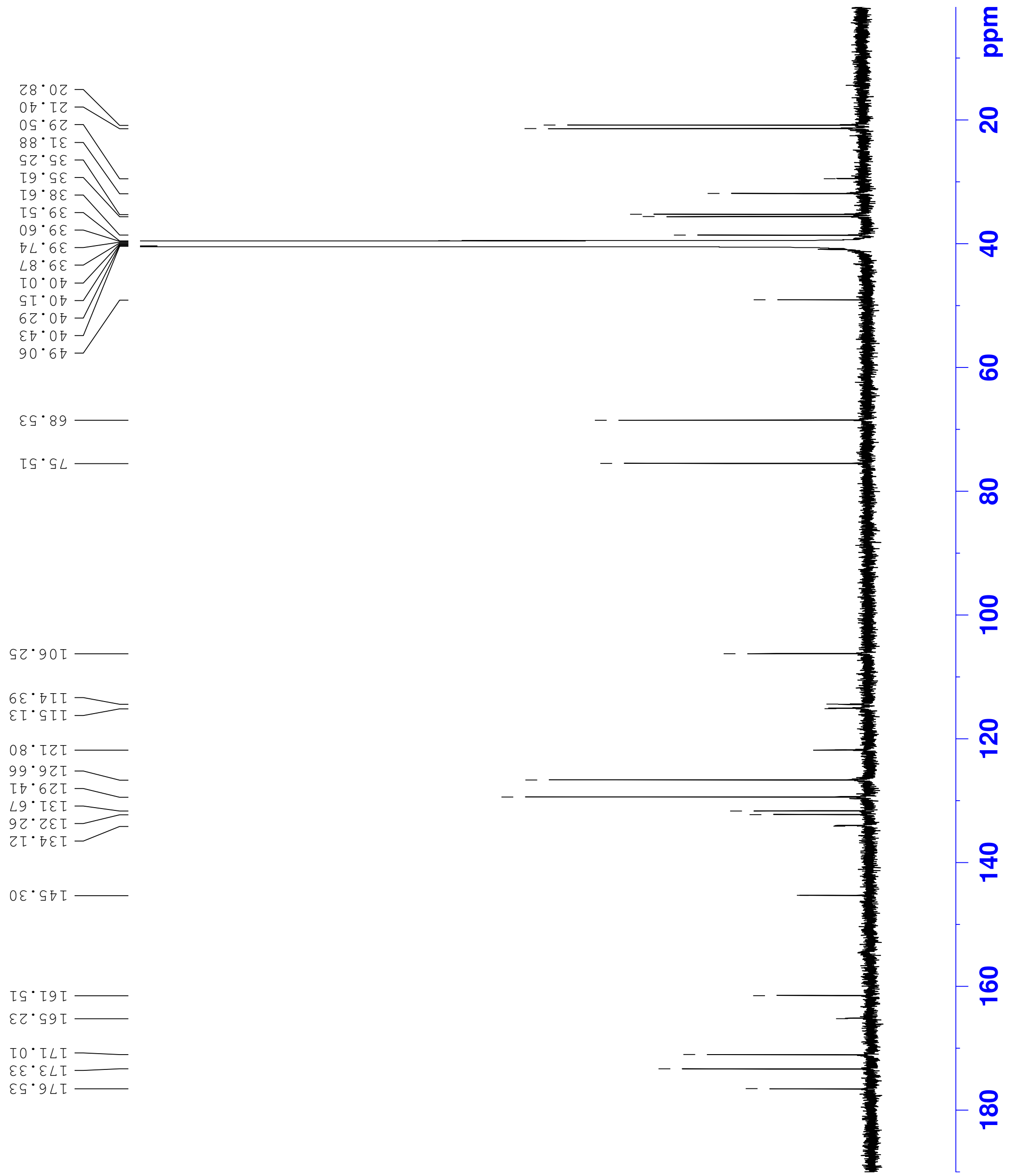

Figure S6. 13C spectrum of the pantetheine-conjugated 7,8-DHF.

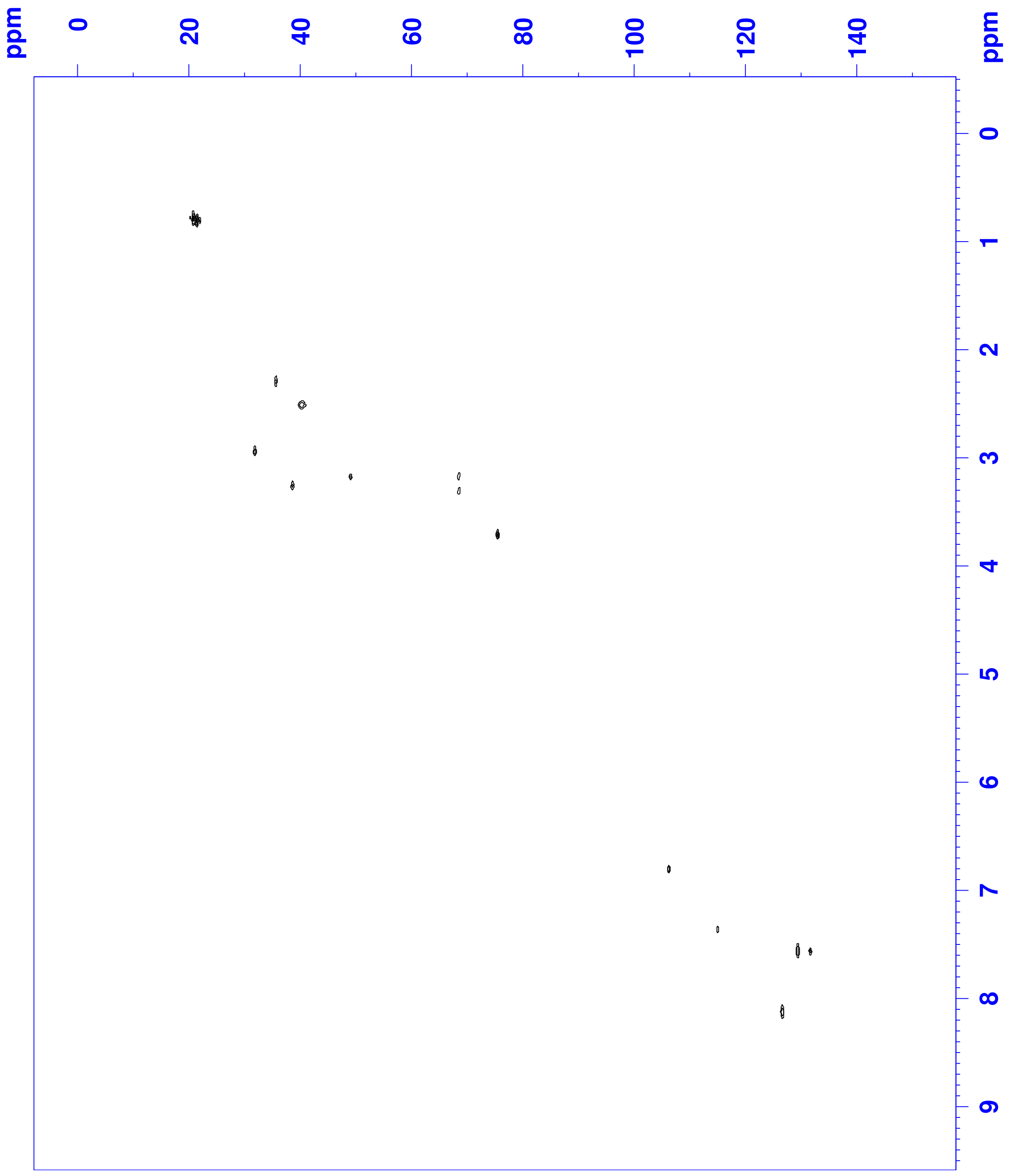

Figure S7. **HMQC spectrum of the pantetheine-conjugated 7,8-DHF.**

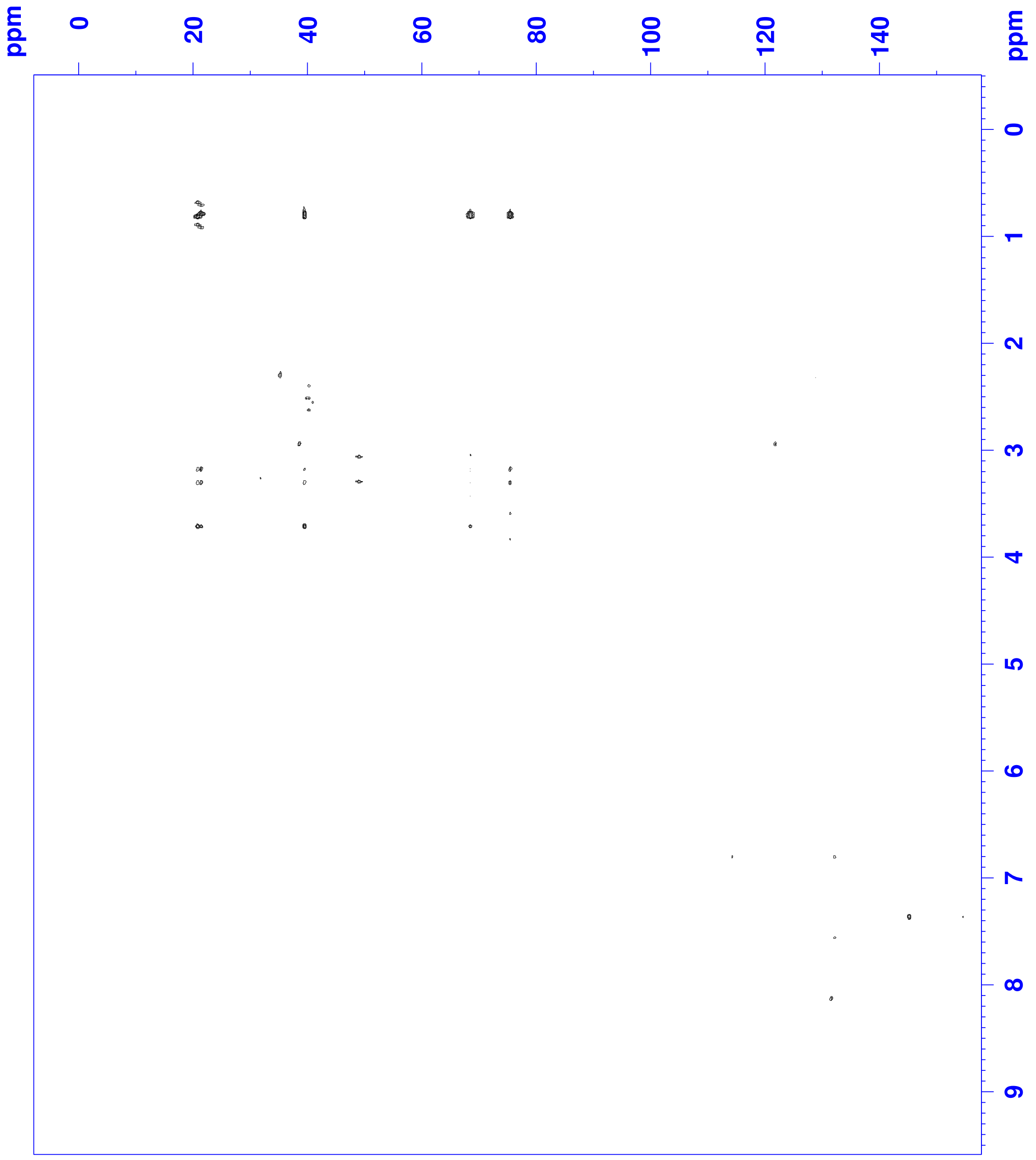

Figure S8. **HMBC spectrum of the pantetheine-conjugated 7,8-DHF.**

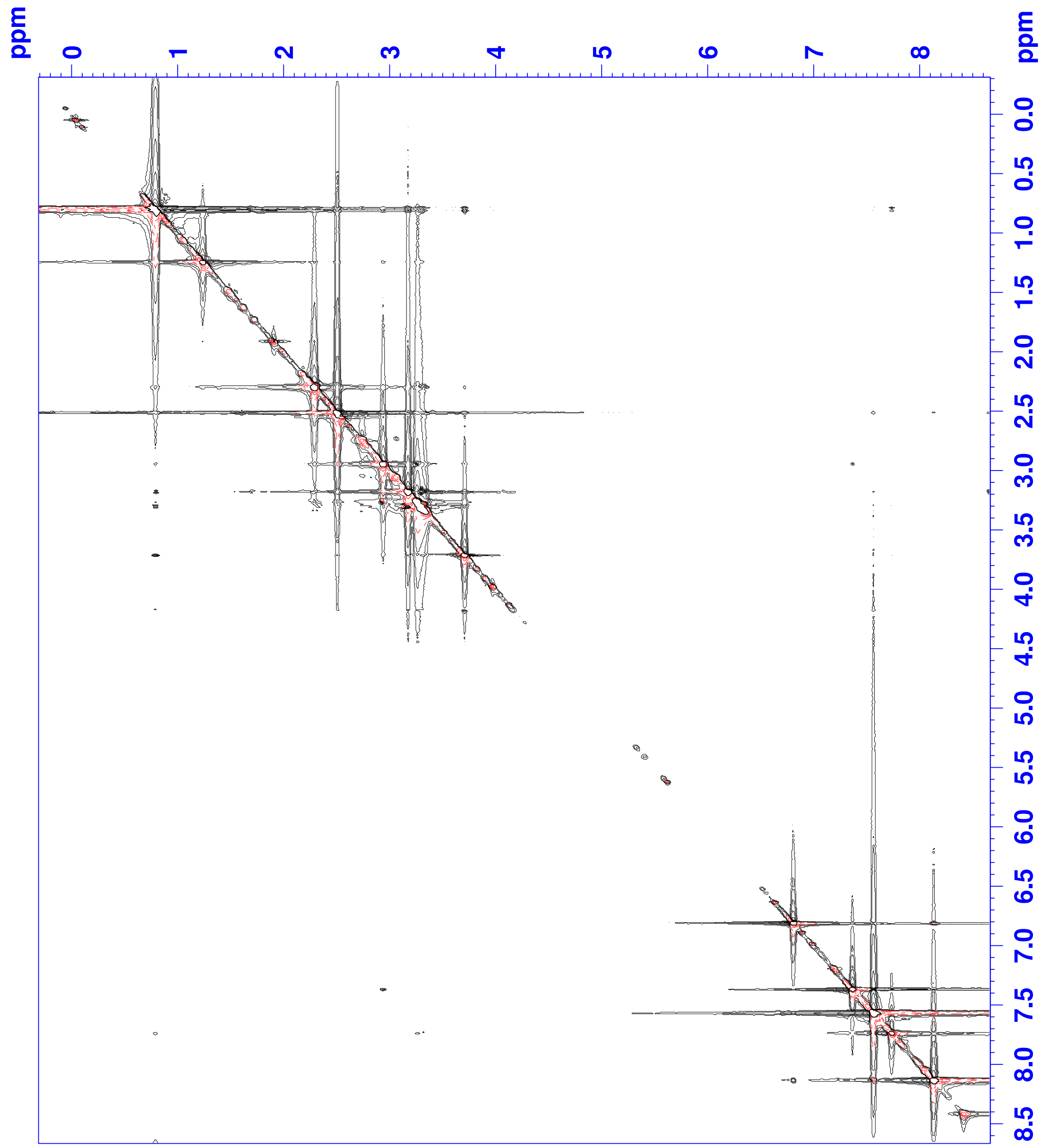

Figure S9. NOESY spectrum of the pantetheine-conjugated 7,8-DHF.

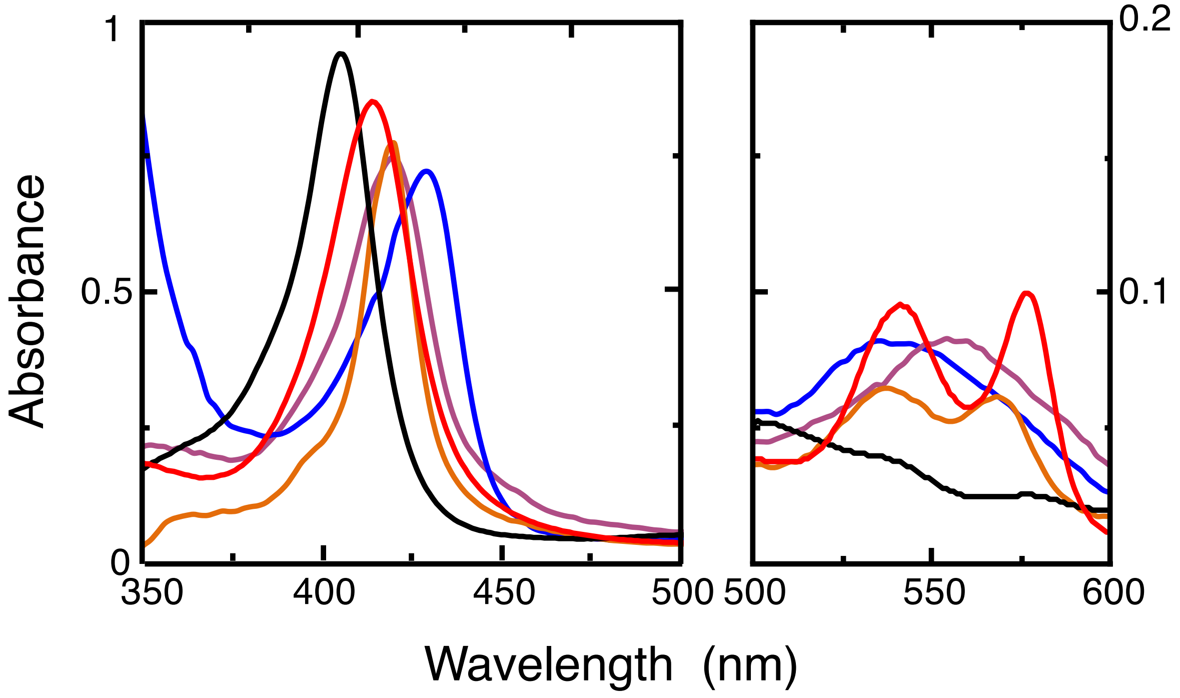

Figure S10. Absorption spectra of Hb on the addition of each of various compounds.

Absorption spectra were measured at room temperature. The final concentration of Hb was 0.225 mg/ml. Black line, $K_3[Fe(CN)_6]$-oxidized; red line, purified Hb; orange line, $K_3[Fe(CN)_6]$-oxidized plus KCN measured under aerobic conditions; blue line, $Na_2S_2O_4$-reduced; purple line, $Na_2S_2O_4$-reduced plus CO measured under anaerobic conditions. The final concentrations of $K_3[Fe(CN)_6]$ and $Na_2S_2O_4$ were 2 mM.

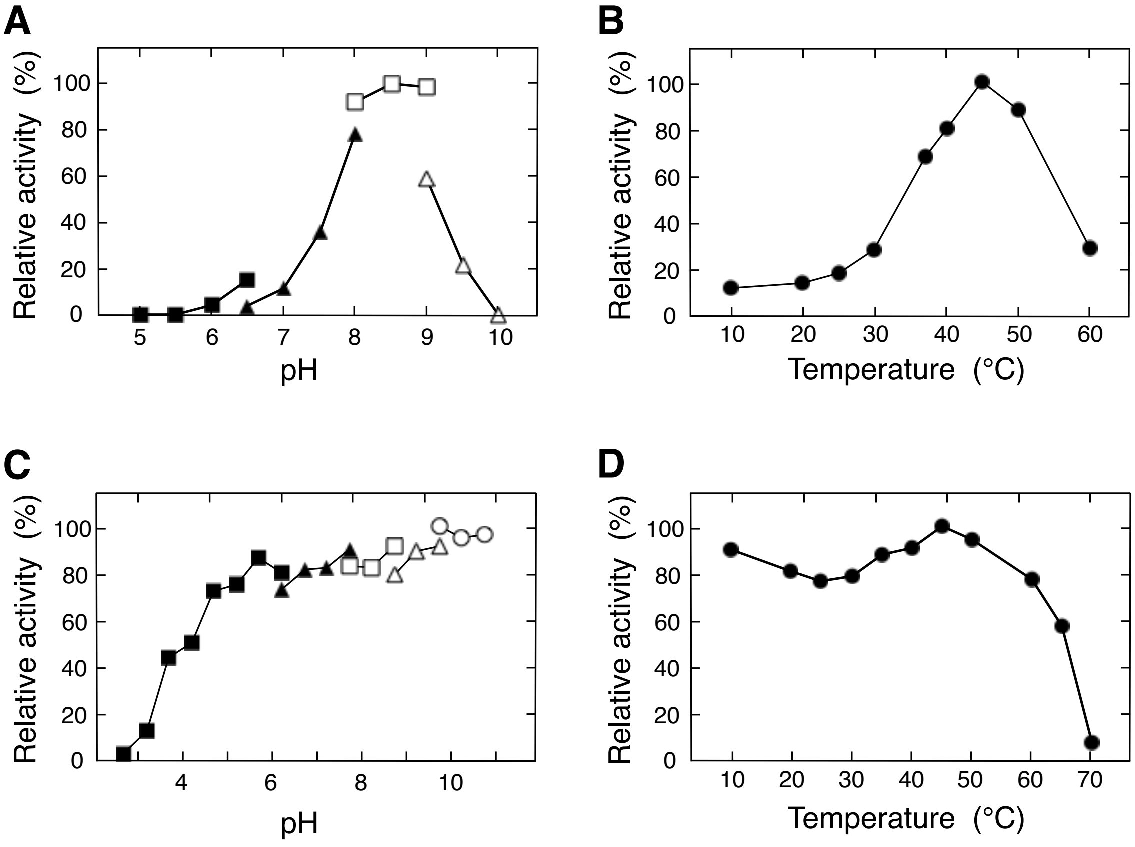

Figure S11. Effects of temperature and pH on the activity and stability of Hb.

A, reactions were carried out for 4 h at 37°C in the following buffers (100 mM): citrate/sodium citrate (▪), Hepes-NaOH (▲), Tris-HCl (▫), and NH_4Cl/NH_4OH (△). *B*, reactions were carried out for 4 h at various temperatures. *C*, Hb was incubated at various pH values at 25ºC for 30 min in the buffers used in *A* and $NaHCO_3$/NaOH (○) at a concentration of 100 mM, an aliquot of each solution was taken, and then the activity of Hb was assayed under the standard assay conditions. *D*, Hb was preincubated at various temperatures for 30 min in 100 mM Hepes-NaOH (pH 7.4), and then the residual activity was assayed. Experiments were carried out three times independently.

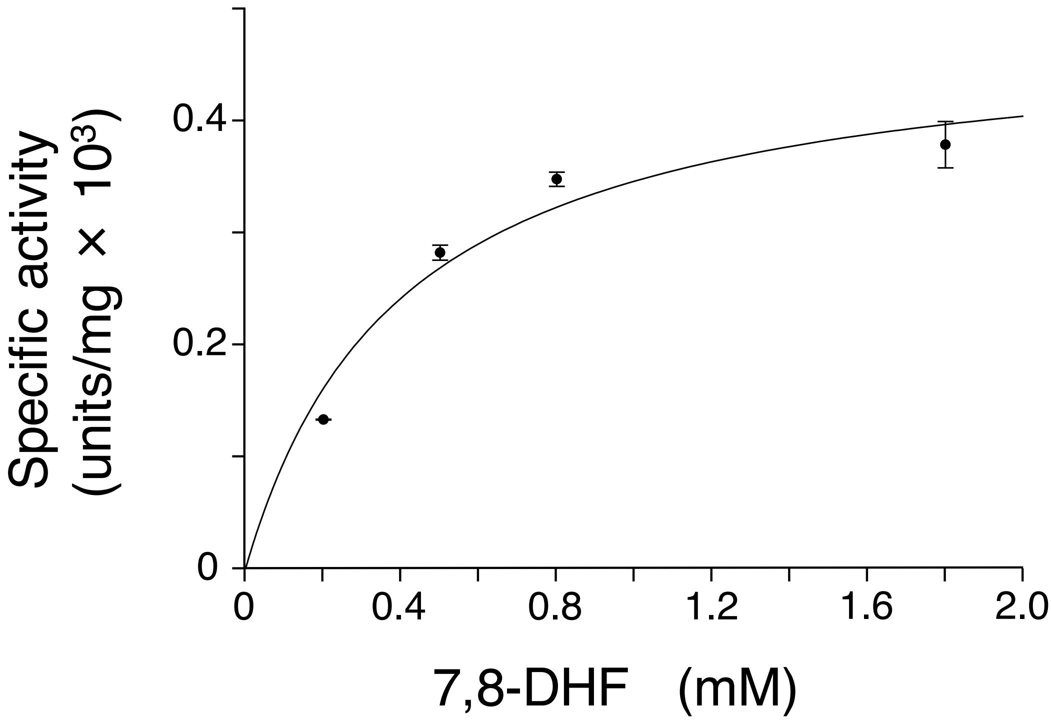

Figure S12. Michaelis-Menten analysis of Hb.

The final concentrations of Hb and CoA were kept at 4 mg/ml and 2 mM, respectively. Experiments were carried out three times independently.

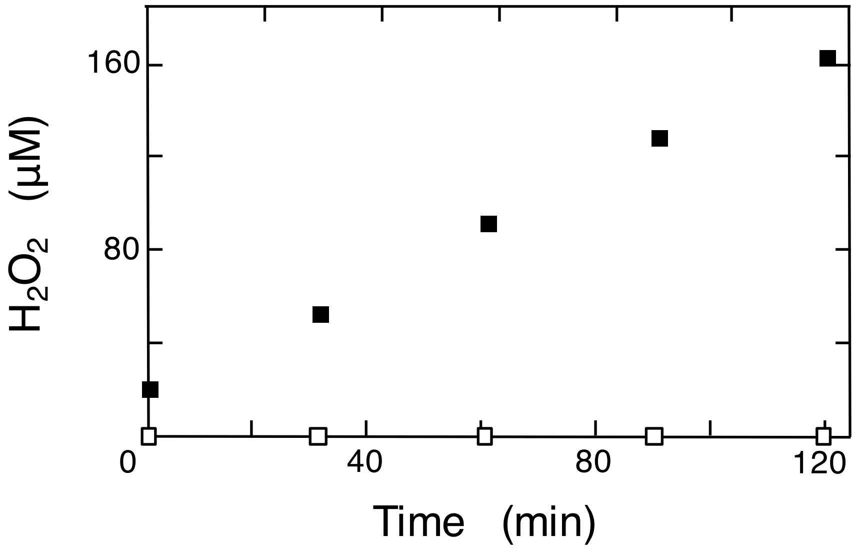

Figure S13. Non-enzymatic and time-dependent production of H_2O_2 in the presence of 7,8-DHF.

The reaction mixture without Hb was incubated at 37°C. H_2O_2 in the reaction mixture was quantified in the presence (▪) or absence (▫) of 7,8-DHF. Experiments were carried out three times independently.
